# In to the feeling: doctoral students’ academic emotional experiences and regulation

**DOI:** 10.3389/fpsyg.2026.1724209

**Published:** 2026-02-12

**Authors:** Yilong Ji, Qunfeng Gui, Gaoyu Chen, Yong Cui, Siyuan Chen

**Affiliations:** 1 Laboratory Construction and Equipment Management Office, Zhejiang Normal University, Jinhua, China; 2 College of Education, Zhejiang Normal University, Jinhua, China; 3 College of Child Development and Education, Zhejiang Normal University, Hangzhou, China

**Keywords:** academic emotion, doctoral students, emotional experiences, emotional regulation, emotional states

## Abstract

**Introduction:**

The process of doctoral students engaging in academic research involves a series of positive and negative academic-related emotional experiences, to cope with the change in emotional states, the use of emotional regulation strategies is encouraged. Although inseparable, few scholars have linked the two for explorations. This study explored Chinese doctoral students’ academic emotional experiences and the corresponding emotional regulation strategies, especially the potential links.

**Methods:**

A total of 15 in-depth interviews were applied to encode and interpret the emotional context of doctoral students. The analytic process was informed by a qualitative interpretive paradigm according to the operation steps of the grounded theory.

**Results:**

The study found that Ph.D. students reported mixed emotions in doctoral programs and supervisor-student interactions, and evident negative emotions in academic writing and academic environment. Furthermore, four emotional regulation strategies were identified to cope with changes in emotional states linked to academic emotional experiences: presupposition, cognitive conversion, restraining, and physiological modulation.

**Discussion:**

The study explores the emotional experience characteristics and emotion regulation strategies of doctoral students throughout their academic period. Links between academic emotional experiences and emotional regulation are highlighted from both relational and context-level perspectives. This not only fills the gap in the study of the relationship between academic emotion experiences and regulation, but also provides a practical basis for reforming doctoral training in terms of ways and environment.

## 1 Introduction

Doctoral students engaging in academic research is a process of pursuing rationality, accompanied by various academic emotions (Villavicencio and Bernardo, 2013; Tan et al., 2021). Academic emotions of doctoral students represent a complex psychological state, with the core being the continuous ehancement or waning of academic interest (i.e., the enhacing or weakening of intrinsic motivation for academic research). The academic emotional issues that Chinese doctoral students encounter during their studies are deeply rooted in the unique social culture, higher education system, and evaluation system of China. They mainly manifest in several aspects: the predicament of academic identity recognition, the alienation and depletion of academic interests, and the institutional pressure and moral burden. Firstly, Chinese doctoral students struggle and have self-doubts between their identities as “scholars” and “students” when stuck in academic identity recognition (Han and Xu, 2023; Geng and Yu, 2022). Especially in interdisciplinary fields, the lack of a sense of belonging and academic community support among students (Deng et al., 2019) is caused by the insufficient support for the “scholar” identity of doctoral students and the ineffective organizational coordination of interdisciplinary cultivation, resulting in a blurred identity recognition (Gu et al., 2018). However, the Chinese doctoral student cultivation system emphasizes the unity of “title” and “content,” which students share the tittle of doctoral candidates while struggling with actual content should be mastered to meet with the standards and requirements as a researcher, and the identity ambiguity leads to deep anxiety. At the same time, the collectivist culture has high expectations for integrating into the academic community, and the sense of isolation is thus magnified (Li and Xue, 2022). Secondly, the weakening of academic interests from enthusiasm to passive coping, as well as the anxiety of exploration (Sverdlik et al., 2018; Han and Xu, 2023) are the adverse results of excessive pursuit of efficiency and “quantitative” achievements, and thus lead to the alienation and depletion of academic interests. However, the Chinese pragmatism tradition and the “result-oriented” nature of doctoral student cultivation have further amplified this conflict and anxiety (Gu et al., 2018; Li and Xue, 2022). Thirdly, the fear of delayed graduation, the pressure of rigid publication, and the unbalanced supervisor-student relationship (Han and Xu, 2023; Geng and Yu, 2022; Gu et al., 2018; Li and Xue, 2022) are common source of the institutional pressure and comprehensive manifestations of the cultivation system’s lack of flexibility, single standards, and excessive concentration of the supervisor’s power (Han and Xu, 2023; Geng and Yu, 2022). However, the strict expectations for life stages and the power distance of respecting teachers (influenced by Confucian culture) are undeniable moral burden in real situations (Gao, 2008; Han and Xu, 2023; Geng and Yu, 2022). The issues of negative academic emotions requires urgent attention. It is noticed that negative academic emotions often accompanied by emotions such as anxiety and self-doubt. These psychological perceptions can transform into external behavioral tendencies and negative attitudes (such as reducing research input) and actual escape (such as considering dropping out or giving up an academic career) (Jackman et al., 2022; Devos et al., 2017). Consequently, doctoral students face a severe mental health crisis, specifically characterized by widespread anxiety and depression (Woolston and O’Meara, 2019). The existing research on the emotional state of doctoral students mainly focuses on the following aspects: (1) emotional types or problems (Geng and Yu, 2022; Han and Xu, 2021); (2) The source of a particular emotion (Anttila et al., 2021; Geng and Yu, 2022; McAlpine and McKinnon, 2013); (3) Strategies for regulating specific emotions (Geng and Yu, 2022; Mahfoodh, 2017). The study of doctoral students involves the transition of professional and academic roles from dependent researcher to independent researcher (Laudel and Gläser, 2008), academic emotional experience runs through the whole doctoral career. It is worth noting that in the existing research on doctoral students’ academic emotional experiences, especially in the regulation of emotions, the combined influence of emotional relationships (such as supervisor guidance) and emotional environments (such as educational system and culture) is often overlooked (Geng and Yu, 2022; Han and Xu, 2021; Tsang and Jiang, 2018). In this case, the lack of relational perspective in emotion regulation may lead to the pure use of strategies rather than the enhancement of emotional competence (Grandey and Gabriel, 2015). This is undoubtedly detrimental to the academic career of doctoral students. It is particularly necessary to focus on the complete academic emotional experience of doctoral students and to view emotions and situations from a relational perspective. Therefore, to clarify the sources of doctoral students’ emotional experience, and explore the internal correlation between emotional experience and emotional regulation for emotional changes in relationship states is particularly important.

### 1.1 Emotional dynamics in academic experience

#### 1.1.1 Definition of academic emotional experiences

Emotional experience is not only the emotion itself but also the process of emotional change in specific situations. According to Pekrun et al. (2002), emotions related to success and failure experienced in academic situations are academic emotions. Similarly, Gross (2015) pointed out that the selection and change of the situation will significantly affect individual emotional states. More importantly, Watson et al. (1988) viewed the characteristics of individual emotional states from the perspective of positive and negative emotions, and proposed an analytical framework of emotion typification. On this basis, a large number of studies explored the emotional state of doctoral students from the perspective of emotional types and showed mixed emotions in the process of learning according to the differences in academic situations (Morrison-Saunders et al., 2010; Mahfoodh, 2017; Anttila et al., 2021; Geng and Yu, 2022). In addition, studies further reveal the prominent role of positive emotions in individual academic advancement. For instance, positive emotions (such as enjoyment, pride, and happiness, etc.) are found to improve academic achievement and learning engagement, and contribute to the smooth completion of doctoral studies with healthy physical and psychological development (Pekrun et al., 2002; Hidi and Renninger, 2006; Ruthig et al., 2007; Trigwell et al., 2012; Mega et al., 2014). It’s worth noticing that the academic emotions of doctoral students refer to the inner feelings during the process of learning with a focus on emotional experience and expression (Anttila et al., 2021). This indicates that individual academic emotions not only come from emotional experience but also reflect in emotional response and coping with emotional experience.

#### 1.1.2 Emotions in situation: location of emotional experiences

It can be seen that emotional experience has a guiding effect on individual emotional states by associating with emotional situations. However, in addition to the type of emotions and single emotional events, existing studies have paid little attention to the overall academic emotional experience of doctoral students. For instance, academic writing has been revealed as a major situational source of negative academic emotions (Gearity and Mertz, 2012; Cotterall, 2013), while collaborative writing on the other hand elicits positive emotions (Ferguson, 2009). In general, emotional experience is not only concerned with the type of emotions but also closely related to the specific emotional event or situation (Cotterall, 2013). More importantly, The emotional experience of doctoral students runs through the whole learning process. Therefore, the existing research on emotional experience has the following shortcomings: First, the current research pays more attention to a single emotional situation, while ignoring the complete emotional experience throughout the doctoral study. Second, studies tend to focus more on emotions and ignore the influence of situational cues. It can be seen that the analytical perspectives of contexts and interaction are crucial to the in-depth exploration of emotional experience.

### 1.2 Respond to emotions: regulation in emotional experiences

#### 1.2.1 Definition of emotional regulation

Emotion regulation is not only the emotional changes of individuals but also the interaction process with emotional situations. Emotion regulation refers to the process in which individuals activate influences to regulate their internal experiences, physiological responses, and behavioral performance to achieve the goal of emotion regulation (Gross, 2015). As mentioned earlier, emotional experience involves changes in emotional states, and positive emotions have been proven to enhance an individual’s psychological wellbeing while effectively promoting academic progress (Pekrun et al., 2002; Hidi and Renninger, 2006; Ruthig et al., 2007; Trigwell et al., 2012; Mega et al., 2014). Therefore, in the process of promoting positive emotions and avoiding negative emotions, emotion regulation plays an important role in promoting individual emotional and psychological wellbeing. Taking the situation as the trigger point, Gross (1998) proposed a process model of emotion regulation, which is not a single, delayed response, but rather a series of continuous activities that occur throughout the entire process of emotion generation. Individuals intervene at different points along the emotional response trajectory. Specifically, two categories for emotional regulation strategies were identified: antecedent-focused strategies and response-focused strategies. Antecedent-focused strategies applied before the complete formation of an emotional response, and include: (1) situation selection (choosing or avoiding specific situations), (2) situation modification (actively changing the current situation), (3) attentional deployment (guiding attention, such as distraction), and (4) cognitive change (re-evaluating the situation to alter its emotional significance). In contrast, response-focused strategies applied after the emotional response been triggered, and the main manifestation was (5) response modulation (change of emotional responses, such as expressive suppression). Based on this construct, Geng and Yu (2022) identified four types of emotion regulation strategies in doctoral student academic writing: task-related regulation, cognitive change, co-regulation, and attention deployment. However, the emotional regulation process of Gross (1998, 2015) mainly focuses on the emotional changes of the individual and ignores the interaction between the situation and the individual. Previous studies on emotional experience have confirmed that the changing of situations and interpersonal interactions have a significant impact on individual emotional states (Gearity and Mertz, 2012; Cotterall, 2013; McAlpine and McKinnon, 2013). Therefore, emotion regulation may have specific sociological significance due to the influence of context and interaction.

#### 1.2.2 Regulation in relation: location of emotional regulation

Emotional regulation involves interactions between individuals and context and therefore reflects the interaction between the two. Based on the influence of context and the analytical perspective of interactionism, Grandey (2000) proposed that individual emotional regulation (emotional labor) is an act that may involve cognitive transformation in interaction (deep acting), or the ignorance of true feelings to meet the needs of the environment (surface acting). Specifically, emotional labor is a comprehensive process of emotional requirement, emotional regulation, and emotional performance (Grandey and Gabriel, 2015). This perspective of emotion regulation gives special sociological significance to both the individual and the context. On this basis, Zhang et al. (2020) explored early childhood teacher’s emotional regulation strategies and identified four strategies for the coping of professional context: disguising, restraining, self-persuading, and releasing. It is worth noting that these four strategies are reflected in professional situations with professional attributes. For doctoral students, there is little research to explore the impact of situational and interactive characteristics on individual emotional regulation processes. Emotion regulation is not only internal, the support or hindrance of the environment determines the complexity of the development process (Grandey and Gabriel, 2015). Therefore, emotion regulation from the perspective of relationships is crucial to the understanding of doctoral students’ academic emotions.

### 1.3 Culture and emotional states

Emotional experience and regulation in doctoral academic life are inseparable. Especially, the process is in dynamic change based on the characteristics of context and interaction, and therefore affect individual emotional states (Cotterall, 2013). It is worth noting that individuals in diverse cultural backgrounds may have completely different emotional experiences during similar emotional events. For instance, previous research has pointed out that academic writing is positively correlated with negative emotions in doctoral students (Gearity and Mertz, 2012), while collaborative writing induces positive emotions and relieves negative emotional states (Ferguson, 2009). It seems that the collaborative writing functions as positive emotional event when individual suffer from negative ones (such as academic writing). However, the actual context may be different in cultural perspective. According to Hofstede (1980), Western countries believe in individualism and focus on the realization of individual values, while Asian countries such as China believe in collectivism, where common interests may be put above individual choices. In such case, collaborative writing may reduce the emotional burden of individuals for the realization of common interests, or it may aggravate negative emotional experience with the conflict of personal values. Similarly, previous studies have confirmed that the closeness of the relationship between doctoral students and their supervisors is positively correlated with the positive emotional state of the individual (Gearity and Mertz, 2012; Cotterall, 2013; McAlpine and McKinnon, 2013). However, in China, Confucian culture emphasizes the high status of teachers (Gao, 2008), while cultural research on power distance also implies the authority of Chinese tutors in the relationship between teachers and students (Hofstede, 1980). This implies that for the sake of a close teacher-student relationship, doctoral students may suppress or even ignore their true feelings when cooperate with their supervisors, putting the emotional needs of their supervisors before their personal needs, which is not conducive to the maintenance of positive emotional states (Grandey and Melloy, 2017). Therefore, emotional states are not always consistent when individual characteristics affect interactive relations. Existing studies focus on a single emotional situation and response, not only ignoring the complete emotional experience of doctoral students, but also reflecting individual emotional states in static environments. However, the introduction of culture in the perspective of interaction provides different levels of explanation (level of context) for the changes of individual emotional states, which has outstanding practical significance.

### 1.4 The present study

As for the characteristics of doctoral students’ emotional states, the existing studies mainly focus on the types of emotions or the influence of a single situation, ignoring the relationship between the situations (especially the multiple situations in the complete academic life) and emotional states. The purpose of this study is to take positive and negative emotional states as the analytical framework, focus on the internal correlation between the complete academic context and emotional states, and explore the characteristics of doctoral students’ emotional experience in academic life, in order to fill the research gap of doctoral students’ emotional states from the perspective of relationship. In addition, as for the emotion regulation strategies of doctoral students, existing studies mainly focus on emotion itself, while ignoring the exploration and analysis of interactive relationships (environment or interpersonal). From the perspective of sociology, the emotional regulation process of interactive relationships involves not only the emotional environment and individuals but also the emotional changes under the mutual influence of the environment and individuals (Grandey and Melloy, 2017). Therefore, this study aims to explore the emotion regulation strategies of doctoral students in the process of emotional experience from the perspective of interactive relationship analysis and strives to make up for the deficiency of emotion regulation strategies under a single analysis perspective (individual emotions or a single situation). Based on this, this study raises the following questions:

(1)    What characteristics do doctoral students’ academic emotional experiences present in academic life?
(2)    What emotion regulation strategies do doctoral students use to cope with changes in emotional states during emotional experiences?

## 2 Materials and methods

### 2.1 Participants

Given the complexity and privacy of emotional experiences during doctoral study, it is of particular significance for research to approach this complex human experience through the narrative of the actors. Following a multiple case studies design with each student as a case embedded within different institutions. Purposive sampling was adopted with the following criteria: (1) The participants had been admitted to the doctoral program and were in the process of studying for a degree. (2) Participants should either have research experience or were still in the process of carrying out research. (3) Participants were willing to share their emotional experiences in academic research. To avoid the potential influence of regional and disciplinary differences (between the humanities and social sciences and science and engineering), we invited current doctoral students from different regions and disciplines through anonymous online video interviews. Therefore, through online invitations and snowball sampling, according to the differences in rankings (national rankings) and academic completion, 20 doctoral students who suffering from emotional distress during studies from top universities and general universities were invited to participate in the study at three different stages: the beginning, middle, and end of their studies. Participants were informed in detail about the study’s purpose, requirements, precautions, and consent forms, and 15 Ph.D. students agreed to participate the interview (see Table 1). Among them, there are six male doctoral students and nine female students from six universities located in Beijing, Shanghai, Zhejiang, and Jiangsu provinces, all with good SES and education quality. In addition, nine of the participants were in the field of humanities and social sciences, and the other six in science and engineering.

**TABLE 1:** Basic information for interview participating doctoral students.

No.	Gender	Grade	Contact channel	Areas of expertise	No	Gender	Grade	Contact channel	Areas of expertise
IV-1	Female	First year	Snowball sampling	Humanities and social sciences	IV-9	Male	Second year	Snowball sampling	Humanities and social sciences
IV-2	Male	Third year	Snowball sampling	Humanities and social sciences	IV-10	Female	Second year	Snowball sampling	Science and engineering
IV-3	Female	Second year	Network invitation	Science and engineering	IV-11	Female	Third year	Network invitation	Science and engineering
IV-4	Male	First Year	Snowball sampling	Humanities and social sciences	IV-12	Female	Second year	Network invitation	Humanities and social sciences
IV-5	Male	Second year	Snowball sampling	Science and engineering	IV-13	Female	First year	Network invitation	Humanities and social sciences
IV-6	Female	Third year	Network invitation	Science and engineering	IV-14	Male	Fourth-year	Snowball sampling	Humanities and social sciences
IV-7	Female	Fourth year	Network invitation	Humanities and social sciences	IV-15	Female	First year	Network invitation	Science and engineering
IV-8	Male	Third year	Snowball sampling	Humanities and social sciences	–	–	–	–	–

### 2.2 Data collection and analysis

#### 2.2.1 In-depth interview

In response to the emotional experiences of doctoral students in academic learning, in-depth semi-structured interviews were conducted to explore doctoral students’ emotional regulation strategies in order to cope with emotions in the emotional experiences. During the interview, special attention was paid to the emotional experiences that trigger changes in positive and negative emotional states and coping strategies as well as support needed. Participants were encouraged to share views on questions such as “How do you describe your emotions in different learning stages?” “What makes you feel negative during your study?” “How did you regulate such emotions?” “Do you think you are supported well?” Each interview lasted around 60 min and was conducted in Chinese and recorded with a permit. The interview records were kept confidential and anonymous to protect the interviewees’ privacy and then transcribed verbatim and sent back to participants for accuracy check.

The analytic process was informed by a qualitative interpretive paradigm (Miles and Huberman, 1994). Data analysis was mainly conducted by Nvivo12.0 according to the operation steps of the grounded theory: open coding, axial coding, and selective coding. First, the description that might lead to the answer was highlighted and encoded into the initial category during the open coding process. After that, the initial codings were compared, merged, and reclassified. Finally, according to the relevant literature, the logical relationship between the codes should be generalized to form the overreaching category (see Table 2; Miles and Huberman, 1994). In order to test the external validity of the coding system, analytical analogy, and participant test are two feasible methods. On the one hand, three doctoral students outside the participating group were interviewed and answered the above questions. No new concepts and categories were found after analysis. On the other hand, the research results were reported back to the five doctoral students who were not involved in interviews, and no obvious misunderstandings were found.

**TABLE 2:** A sample of data coding steps.

Research issues	Research object	Opening coding	Axial coding	Selective coding
Emotional experience of doctoral students	IV-2	After the paper was published, I felt very happy and proud. (good feeling)	Motivational emotion	Positive emotion
Emotion regulation strategies of doctoral students	IV-15	Not every publication is successful, and most submissions fail. But we need to calm down. (emotional control)	Situation selection	Presupposition

IV, interview. The highlighted part refers to the interaction between doctoral students and different academic activities. The underlined part refers to doctoral students’ emotional experience or emotional regulation strategy in academic activities.

## 3 Results

### 3.1 Characteristics of academic emotional experience

According to the analytic process, there are four major sources of doctoral students’ academic emotional experiences: doctoral programs, academic writing, supervisor guidance, and academic environment. The distribution of positive and negative emotional experiences reflects the overall patterns and characteristics (see Table 3). Doctoral students showed mixed feelings and emotions with slight differences in frequencies toward doctoral programs (7.7% and 5.2%) and supervisor guidance (13.2% and 10.8%). This indicates that doctoral students generally recognize the process of learning and have a positive attitude toward possible gains. At the same time, the arduous process of learning and research can also cause stress and a range of negative emotions, and doctoral study is generally a mixed blessing.

**TABLE 3:** Distribution of academic emotions during doctoral study.

Critical academic events	Type (P + N)	Positive academic emotions (P)	Frequency	Negative academic emotions (N)	Frequency
Doctoral programs	8	Excitement, satisfaction, interest, expectation, gratitude	22 (7.7%)	Tension, frustration, boredom	15 (5.2%)
Academic writing	20	Happy, excited, surprise, love, enjoy, satisfaction, pride, release	47 (16.4%)	Depression, anxiety, stress, sadness, loneliness, anger, disappointment, confusion, fatigue, dissatisfaction, frustration, boredom	83 (28.9%)
Supervisor guidance	13	Care, gratitude, moved, satisfied, proud, positivity, warm	38 (13.2%)	Disappointment, fear, grievance, passivity, inferiority, guilt	31 (10.8%)
Academic environment	10	Happy, calm and firm	9 (3.1%)	Anxiety, stress, loneliness, helplessness, disappointment, dissatisfaction, boredom	42 (14.6%)
Total	38	–	116 (40.4%)	–	171 (59.6%)

The distribution of academic emotions during doctoral study are categorized into positive and negative emotions with frequencies to show the variations among critical academic events.

IV-13, Dr. Wang: I have been working for 6 years, and I am very excited to go back to school again to do my Ph.D. study. The Ph.D. program is very different. The teacher will let us go to the seminar and debrief ourselves each time. I have learned a lot. However, every time I get to the end of the semester, I get nervous I still don’t know how much I have done according to the plan.

On the other hand, interaction between the supervisor and the student is concentrated in the discussion of research issues and the supervision of research progress. Therefore, the progress and stagnation of the research may trigger positive or negative emotional experiences, and it is worth noting that the expression of emotions is more significant in the interaction.

IV-14, Dr. Lee: I am very grateful to my supervisor for his help and support during my Ph.D. study. When I encounter difficulties in writing or daily life, I always communicate with my supervisor, while if any progress in academic research, I will immediately share my joy as well. (05-2024)

IV-10, Dr. Li: This semester, my supervisor is too busy with administrative affairs. Whenever I try to discuss on my research, I can not reach him. I feel disappointed. (05-2024)

It is worth noting that academic writing is considered to be a significant source of stress and negative emotional experience for doctoral students, mainly manifested in more emotional types and higher emotional frequency (12 negative emotions and eight positive emotions; 28.9% and 16.4%). Academic writing is not only the writing itself, but also involves a series of complex and rigorous processes such as research questions, research design, research methods, data collection and analysis, and comprehensive discussion. When problems occur at any part of the process, it can lead to negative emotional experiences as a result.

IV-4, Dr. Duan: I remember when everyone was preparing for the doctoral dissertation, and I stuck at my experiments with unsatisfactory outcomes. As a result, I have to postpone the opening. I felt particularly stressed and afraid for graduation. (04-2024)

In addition, academic writing also involves the publication required for graduation, and students who fail to publish in journals of certain quality during their studies may face the problem of postponing graduation or even dropping out.

IV-7, Dr. Sun: The reason why I suffer from depression is that during the third academic year, the paper has not been published, it was painful, even hesitating on quit. It becomes worthless and the quality of my sleep is alarming. (03-2024)

At the meantime, according to the results, academic environment is very disappointed to doctoral students. Much more frequency of negative emotions are showed compared to the positive ones (14.6% and 3.1%). First of all, although independent researchers, the academic environment of Chinese doctoral students is more competitive than collaborative. This pressure of peer competition may lead to a great amount of negative emotional experiences.

IV-2, Dr. Shang: Some of my classmates started the topic in June, but I have no progresses at all. What makes me particularly anxious is that the male doctors in our subject group often ask me about the progress of my thesis, and he has published two SCI articles. What’s more hateful is that someone will steal your innovative ideas. (04-2024)

Secondly, the academic training system may obtain high requirements for doctoral students. On one hand, the thesis is expected to be creative and qualitative, so that it may serve the future academic career. On the other hand, publications are required to show abilities and accomplishments of doctoral students, so that it identify the graduation and reputation.

IV-11, Dr. Xie: I have to be creative with good research questions and diverse methods to testify the hypothesis. Also, university requires at least 2 SCI papers for graduation. Every day, I feel precious, always worried that I won’t be able to graduate at last. (04-2024)

### 3.2 Academic emotion regulation strategies

According to the results, there are four major emotion regulation strategies for the managment of emotional experiences: presupposition, cognitive conversion, restraining and physiological modulation.

#### 3.2.1 Presupposition

Presupposition is a doctoral student’s conscious presupposition of situations that may trigger emotions. When individuals choose positive situations, they are more likely to induce positive emotions. At the same time, individuals consciously choose to avoid negative situations in order to achieve the purpose of emotional control.

IV-9, Dr. Mo: Getting published wasn’t as easy as I thought. I could be prepared and focused on the process rather than the outcome. (03-2024)

When it comes to communicating with supervisors, in order to avoid negative emotional atmosphere, doctoral students are often inclined to report the recent research progress first, and then raise the problems encountered.

IV-4, Dr. Duan: I made an appointment with my mentor to meet and discuss in the afternoon. I need to sort out what I have done recently and make him aware of my efforts. That way, he might not get angry when I raise a question. (04-2024)

IV-2, Dr. Shang: My supervisor is highly authoritative, but his research approach is rather traditional, and tending to optimize within the existing framework. To gain his attention and approval, I anticipated that directly presenting a revolutionary idea would be hard for him to accept, as he needs to assess risks for graduation. Therefore, instead of merely discussing the idea, I spent 2 weeks crafting a very detailed “preliminary research report.” I collected all the literature supporting this new direction, analyzed the feasibility of the technical path in detail, and even preliminarily designed a small-scale verification experiment plan, estimating the required time and resources. Surprisingly, the outcome was excellent. Although he still held a reserved attitude, he agreed to conduct this verification experiment. (04-2024)

This episode shows that in the academic life of doctoral students, there may be different situations that induce positive and negative emotions. With the accumulation of experience, doctoral students will consciously anticipate situations, focus on positive situations and avoid negative ones, and finally achieve emotional regulation and management.

#### 3.2.2 Cognitive conversion

Cognitive conversion is an emotion regulation strategy based on cognitive change. It is mainly reflected in that doctoral students assign meaning to the perceived situation by transforming their cognitive perspectives, and re-evaluate the abilities to cope with and manage the situation.

IV-15, Dr. Mu: Sometimes when my tutor scolds me, I feel wronged. I often tell myself that I must put myself in others’ shoes. My tutor is trying to make me excellent by pushing, if I can do better next time, he will be happy. (04-2024)

IV-4, Dr. Duan: Scientific research writing is a tough process. I have experienced countless rejections and revisions. The undercurrents beneath this “calm surface” have brought my work efficiency to a standstill. So, I began to carefully examine and transform my understanding of this interaction. First, I tried to distance myself from the identity of a “loser.” I told myself, “’Rejection is a common occurrence in scientific research, and it doesn’t mean I am a failure as a person.” I separated “my self-worth” from “the outcome of the article.” Secondly, I made an effort to calm down and read the reviewers’ comments repeatedly. At first, I felt that each comment was an attack, while gradually, when I reflected on it from an objective perspective, I found that the reviewers’ comments did indeed point out the weaknesses that had been overlooked or deliberately avoided. (04-2024)

IV-10, Dr. Li: My doctoral thesis relies on a crucial synthetic experiment, which is theoretically feasible and my supervisor is full of expectations. However, over the past 400 days, I have repeated the experiment 200 times and adjusted dozens of parameters, but the result has been like being cursed, with the ideal crystal structure remaining elusive. After countless setbacks, I began to try to change my mindset and attitude. This process was extremely painful because it required me to confront “failure” and redefine it. I forced myself not to view these 200 failures as “200 pieces of evidence proving my incompetence,” but rather as “200 data points eliminating wrong paths.” I started to record in detail the conditions and results of each failure, organizing them into a huge “failure database.” I told myself that my work might not be to synthesize the target crystal, but to thoroughly understand “why it cannot be synthesized in this system.” This shift in perspective was revolutionary, transforming my doctoral project from “verifying a success” to “exploring a mystery,” and failure became the most important component of this mystery. (05-2024)

#### 3.2.3 Restraining

Restraining is a strategy used by doctoral students to regulate and manage emotions by suppressing or isolating their inner feelings when they perceive dissonance between emotional display and genuine feelings. Suppression is the first strategy.

IV-7, Dr. Sun: Even if there are times when I feel bad about unfair systems, I don’t vent my anger but rather stay calm. Because anger doesn’t solve anything, it just makes me worse. (03-2024)

IV-4, Dr. Duan: My process of emotional adjustment was a tough “tug-of-war.” I was completely dominated by negative emotions and adopted a passive and indifferent strategy. I tried to pretend that nothing had happened, not thinking about rejection letters, and forced myself to continue with the continuous experiment. But emotions couldn’t be suppressed. When processing the data, I became extremely irritable and lost my usual patience with my junior colleagues. (04-2024)

Isolation is another restraining strategy and doctoral students are likely to apply when there are derived intense feelings and emotions. Instead of being trapped with such situations, they prefer to isolate the emotions by interacting with clam and peaceful environment.

IV-3, Dr. Yuan: During experiments, sometimes I may encounter difficulties that make me feel very frustrated or extremely disappointed. To calm myself down, I look out the window for the view and listen to soothing music for a while. (03-2024)

This episode suggests that restraining is not necessarily a passive emotion regulation strategy, through the stimulation and interaction of positive situations, individuals also actively isolate negative emotions and transform their inner feelings.

#### 3.2.4 Physiological modulation

Physiological modulation is a strategy that doctoral students use to cope with or regulate emotions through physical behavior (such as exercise) or specific physiological habits (such as exercise habits). This adjustment can either be immediate, or habitual.

IV-10, Dr. Dai: Sometimes when I’ve been in the library for a long time, I feel depressed. I like to do exercise on the playground or take a nap. (04-2024)

IV-14, Dr. Lee: When I feel extremely lonely and helpless, I engage in a “physiological adjustment” - my method is to eat. A meal prepared by myself, often helps soothe my academic frustrations and feelings of loneliness. (05-2024)

Doctoral research is a long-term process with mixed feelings and emotions, while long-term physiological modulation function as a compensation for negative emotional experiences.

IV-6, Dr. Ren: Research is hard work for doctoral students and often involves a variety of emotions. Therefore, I take an hour for a nap and another hour for exercise every day to keep myself in a good energy and emotional state. (03-2024)

**FIGURE 1:**
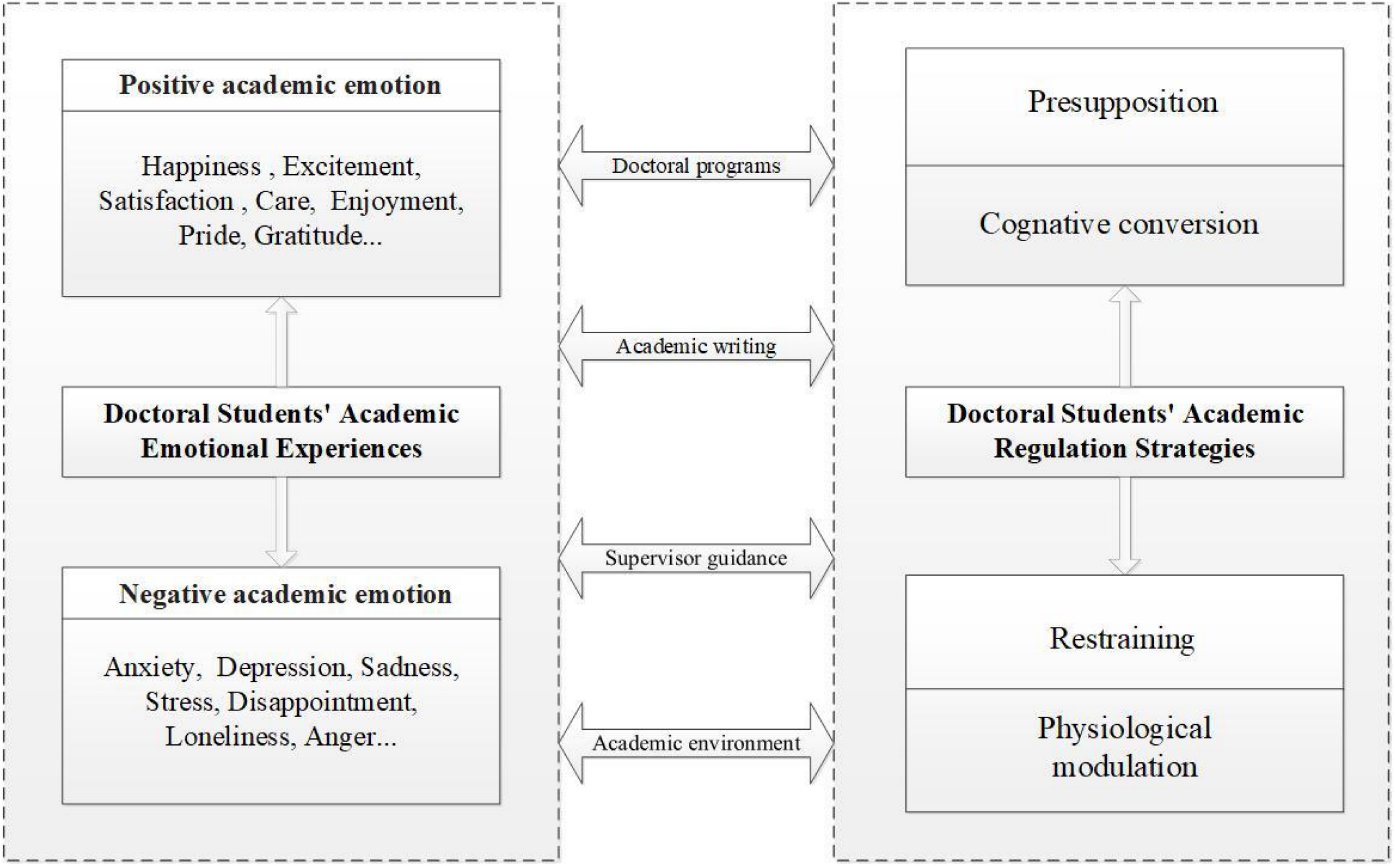
The relationship between emotional experience and regulation in doctoral students.

In addition, it is found that doctoral students experienced both positive and negative academic emotions during critical academic events, with certain emotional regulation strategies based on the relational perspective (see Figure 1).

## 4 Discussion

With the exploration of doctoral students’ emotional experiences in China, this study identified four major sources that trigger emotions from both positive and negative sides: doctoral programs, academic writing, supervisor guidance, and academic environment. According to the results, doctoral students share concerns about supervisor support with few differences in types and frequencies of positive and negative emotions. These results partly echo previous research on the effect of supervisors on doctoral students’ emotions (Stubb et al., 2011; Janta et al., 2014; Sarikaya et al., 2017). First of all, in China’s Confucian cultural system, the principle of respecting teachers and respecting the way is widely emphasized and has far-reaching influence (Gao, 2008). This shows that in every meeting, the supervisor’s attitude sets the tone for the emotional atmosphere of the interaction, and the emotional atmosphere determines the emotional experience of the two interacting parties in the current situation (Devos et al., 2017). In addition, studies have pointed out that the teacher-student relationship in China is not completely equal, and is even affected by the hierarchy (Gao, 2008; Tsang and Jiang, 2018). Similarly, studies on the cultural impact of power distance show that the degree of unequal distribution of power in societies and organizations is closely related to cultural background (Hofstede, 1980). As a result, the authority of the supervisor may lead to students’ mixed emotions, especially based on research progress. Secondly, according to the concept of life-span development, the cognitive resources of individuals in the process of primary control show an inverted “U” shaped development law, and reach the peak in middle age (Baltes et al., 1980). This indicates that compared with the research experience of doctoral students, supervisors have richer cognitive resources and higher expectations for the research itself. That is, supervisors and students are at different levels of cognitive expectations for research. The positive emotions of students may come from the self-affirmation of the research process or the objective adjustment of the supervisor to the research expectation. At the same time, positive emotions may also result from supervisor guidance based on cognitive differences. On the other hand, negative emotional experiences may stem from difficulties in the research process, or emotional conflicts due to cognitive biases.

In addition, the study reveals that during doctoral study, students suffer from academic writing and environment with significantly more negative emotions in types and higher frequencies in performances than positive ones. For doctoral academic writing, there are two major tasks: doctoral thesis and article publication. The doctoral thesis is based on the research question and research design and consists of a series of sub-studies around the research question, each of which also involves the use of methods, the collection and analysis of data, and the discussion of significance. Therefore, from the perspective of composition, doctoral research is bound to go through a long-term argumentation. Some studies have pointed out that this process presents phased characteristics in the emotional response of doctoral students, and with the extension of learning, the cumulative effect of negative emotions is significantly increased (Jackman et al., 2022; Devos et al., 2017). This may be due to the fact that with the progress of thesis research, the complexity of the research and the coping and problem-solving increase with the development of the learning periods, the meantime, negative emotions accumulate within the process (Devos et al., 2017). On the other hand, article publication means standardized requirements for research design, implementation, and writing. Different from a simple course, the writing of a paper is based on the design and execution of the research, which requires continuous thinking and practical exploration of problems in real settings. However, insufficient preparation in the pre-stages may lead to the difficult birth of an article, and therefore individuals show a series of negative emotions. This may also be the reason why doctoral students procrastinate in academic writing (Gearity and Mertz, 2012; Cotterall, 2013). At the same time, academic writing itself requires practice, and procrastination or resistance may have further effects on an individual’s emotional experiences. It is worth noting that the publication of an article undergoes the evaluation of different reviewers from multiple perspectives and dimensions. The waiting period for the review results and the response to the review opinions may induce a series of negative emotions, such as tension or anxiety, and this emotional experience is further highlighted in peer competition.

According to the results, doctoral students experienced more negative emotions in the present academic environment, especially results from peer competitions. Devos et al. (2017) point out that the competition of doctoral students shows a high degree of self-involvement, which is manifested in the comparison of research progress and the quantity and quality of papers published. It is important to note that the competitive state of peers does not represent their willingness to cooperate, especially since successful cooperation may also lead to positive emotional experiences (Ferguson, 2009). Instead, peer competition exacerbates the impact of negative emotions (Devos et al., 2017), and this effect is more significant in the doctoral programs that need to be improved. For example, doctoral students should have more options to prepare for their careers than simply determining the quality of their studies by publication (Yang and Cai, 2022). In addition, in a context where interdisciplinary disciplines are widely recognized, rich learning communities (such as collaboration across research fields or institutions) and academic exchange platforms are also identified as key supportive environments so that doctoral students as independent researchers are less likely to stay alone (Yang and Cai, 2022).

In response to the emotional experiences, this study identified four emotion regulation strategies: presupposition, cognitive conversion, restraining, and physiological modulation. According to the process model of emotion regulation, there are two intervention points for regulation: antecedent-focused, where individuals amend situations or perceptions for emotional adjustment, and response-focused, where physiological or behavioral responses are involved according to the tendency of emotions (Gross, 1998, 2015; Grandey, 2000). In this study, the first two strategies share a focus on antecedents, while the last two strategies tend to deal with emotional responses, and ultimately constitute a complete emotional regulation process. In the study of Gross (1998), individuals can take actions to put themselves in situations that cater to (or avoid) certain emotions. The presupposition strategy in this study not only focuses on individual emotions but also the characteristics of the situation itself, especially the sociocultural connotation of the situation. Specifically, out of respect for the supervisor and awe of the power difference, doctoral students tend to anticipate the supervisor’s discussion expectations before meeting, so that targeted psychological and emotional preparations can be made. This effect may be significant in different cultural contexts, such as in the United States and Europe, where individual competence is valued more than power (Hofstede, 1980).

In addition, in Gross’s (2015) model of the emotion regulation process, individuals alter the influence of emotions by appraisal or reappraisal of the situation, which can be either external or internal. This strategy of cognitive change has also been demonstrated in previous studies on emotional regulation from both sides of supervisors and graduate students (Geng and Yu, 2022; Han and Xu, 2021). However, cognitive conversion in this study shares a focus on relationships instead of concentrating on situations. It is worth noting that when individuals have the goal of emotion regulation, the process of emotion regulation will not only affect themselves but also others. Therefore, the process of emotion regulation includes the internal emotion regulation of individuals (intrinsic emotion regulation) and the external emotion regulation (extrinsic emotion regulation when paying attention to others (Gross, 2015). This indicates that the dynamics of emotional regulation are affected by interaction relations. Especially in this study, instead of cognitive change, cognitive conversion emphasizes the mutual transformation of situational cognition in the relationship and triggers the mutual fitting of emotional states of the two interacting parties. As reported by Dr. Mu (IV-15), the anger of the supervisor may be altered according to better performance. It shows an alteration of self-performance on the side of doctoral students and indicates the possible expectation change on the side of the supervisor for the fit of emotion regulation since there are cognitive differences according to the life-span human development principles (Baltes et al., 1980).

In order to meet the needs of organizations, individual emotion regulation often involves emotional display rules (Grandey, 2000; Grandey and Gabriel, 2015). This shows that emotional regulation is not only concerned with the adjustment process of individuals but is also reflected in the relationship between individuals and the environment. Therefore, inspired by the emotion regulation model, Grandey (2000) proposed an emotional labor model based on the individual emotion regulation process, and emphasized the prominent role of surface acting as a response-focused emotion regulation strategy. Specifically, in addition to cognitive changes, individual emotions also involve a series of adjustment methods that ignore inner feelings, such as suppression and faking. On this basis, Zhang et al. (2020) explored the emotion regulation strategies of preschool teachers and found that restraining is one of the strategies commonly used by teachers. At the same time, restraining also involves the transformation of interactive objects for positive emotional mobilization. Similarly, restraining in this study also involves the suppression and transformation of emotions. Especially when the interaction object is variable and optional, both isolation of negative emotions and conversion of interaction can effectively mobilize positive emotional states. However, the innovation of this study lies in the attention paid to context. Compared with the situation, context could be hierarchical and therefore has completely different effects on individual emotional states. Specifically, although both situation and context represent the environment, individual emotions change with selective situations, while emotional context may remain stable depending on the ecological environment, and even obtain a long-term effect on individual emotional states.

According to Gross (1998, 2015) response modulation exerts influence on the trend of individual emotional response (such as psychological experience, behavior, physiological reaction, etc.) after the emotion is stimulated, which is manifested as an observable reduction or enhancement of emotional response. As part of response modulation, physiological modulation in this study is mainly embodied in two aspects: immediate modulation and habitual modulation. A large number of studies have proved that physiological modulation (such as exercise) relieves and regulates stress and negative emotions (Tsatsoulis and Fountoulakis, 2006; Møller et al., 1996; Bernstein and McNally, 2018; Childs and De Wit, 2014). For doctoral students, the complexity and uncertainty of research affect their emotional states (Jackman et al., 2022; Devos et al., 2017). Immediate physiological modulation can deal with negative emotional trends in a timely manner, while the autonomy of research work and time arrangement ensures the possibility of self-regulation to a certain extent. On the other hand, doctoral research is a long-term process, and studies have pointed out that procrastination leads to a large number of negative emotional experiences (Gearity and Mertz, 2012; Cotterall, 2013). This suggests that planning for research is particularly important during doctoral studies. Therefore, in the process of plan implementation, physiological modulation, as an important factor affecting emotional state, is gradually incorporated into the plan and becomes habitual.

## 5 Conclusion

Different from previous studies that focused on a single academic emotional event (Geng and Yu, 2022; Anttila et al., 2021; Han and Xu, 2021), this study explores the emotional experience characteristics and emotion regulation strategies of doctoral students throughout their academic period through in-depth interviews. First, the complex emotional experiences of interacting with supervisors indicate the significance of the relational perspective compared to the situational perspective. At the same time, the use of emotion regulation strategies such as presupposition and cognitive conversion provides a basis for further revealing the perspective of mutual adaptation processes in teacher-student interaction relationships. This fills the gap in the study of doctoral students’ academic emotions from a relational perspective. Second, the significant negative emotional clustering toward academic writing lays the foundation for studying the stage characteristics of doctoral students’ emotions. Similar to the stage characteristics reflected in academic writing, the emotional state of doctoral students may be closely related to the complexity of research in development (Devos et al., 2017). Meanwhile, the immediacy and habitual characteristics of physiological modulation also reflect the variability of emotional states and the continuity of emotion regulation. Third, the negative emotional attitude toward the academic environment suggests the multiple meanings of environmental and resource support at both the micro (peer competition) and macro (training methods) levels. Furthermore, the use of restraining strategies indicates that when the influence of the academic environment is at different levels, individuals show significant differences in their subjective agency in emotional regulation. This not only fills the gap in the study of the relationship between emotion regulation and the environment but also provides a practical basis for reforming doctoral training in terms of ways and environment.

## 6 Limitation

Based on key emotional events, this study explores the characteristics of doctoral students’ emotional experiences and emotional regulation strategies during their studies. Even if it reflects the emotional state of doctoral students to a certain extent, there are still some limitations in the research. First of all, emotional experience and emotional regulation are not two independent processes. On the contrary, emotional regulation is a complex processing process of emotional states due to the influence of situations or interactive relationships (Gross, 2015; Grandey, 2000). The fit between emotional experience and emotional regulation process in this study is insufficient, especially when doctoral students have significant negative emotions in two typical events: academic writing and academic environment, emotion regulation strategies are not in line with adequate support. More importantly, the interviews on emotion regulation strategies did not conduct in-depth discussions on the influence of the academic environment from both micro (peer competition) and macro (training mode) levels, which may lead to insufficient responsiveness of emotion regulation strategies. Future research can further explore academic emotion around the hierarchical characteristics of the environment on the basis of optimizing the interview framework. Secondly, the characteristics of doctoral students’ negative emotions in academic writing (multiple types and high frequency) may indicate the developmental effects of emotional experience and regulation, especially as the complexity of research increases, individual emotional states may undergo more obvious changes (Jackman et al., 2022; Devos et al., 2017). However, the participants in this study did not show developmental characteristics in their coding (such as differences in years of learning), which may have contributed to the bias in the presentation of the results. Future research can further explore the emotional characteristics of doctoral students in the whole learning process on the basis of incorporating developmental effects into the coding system. Thirdly, the relationship between supervisor and student is of great significance in Chinese culture and the doctoral student cultivation system. In this study, although rich emotional experiences, the relationship in specific emotional situations involved were extremely complex. Due to the prominent position and power distance of supervisor in Chinese culture, future research can further classify the situations and explore the differences and influences on the emotional experiences and regulation of doctoral students under various of relationship patterns.

## Data Availability

The raw data supporting the conclusions of this article will be made available by the authors, without undue reservation.
